# Omeprazole Blocks STAT6 Binding to the Eotaxin-3 Promoter in Eosinophilic Esophagitis Cells

**DOI:** 10.1371/journal.pone.0050037

**Published:** 2012-11-21

**Authors:** Xi Zhang, Edaire Cheng, Xiaofang Huo, Chunhua Yu, Qiuyang Zhang, Thai H. Pham, David H. Wang, Stuart J. Spechler, Rhonda F. Souza

**Affiliations:** 1 Department of Internal Medicine, Veterans Affairs North Texas Health Care System and the University of Texas Southwestern Medical Center, Dallas, Texas, United States of America; 2 Department of Surgery, Veterans Affairs North Texas Health Care System and the University of Texas Southwestern Medical Center, Dallas, Texas, United States of America; 3 Department of Pediatrics, Children’s Medical Center and the University of Texas Southwestern Medical Center, Dallas, Texas, United States of America; 4 Harold C. Simmons Comprehensive Cancer Center, University of Texas Southwestern Medical Center, Dallas, Texas, United States of America; Cincinnati Children's Hospital Medical Center, United States of America

## Abstract

**Background:**

Patients who have esophageal eosinophilia without gastroesophageal reflux disease (GERD) nevertheless can respond to proton pump inhibitors (PPIs), which can have anti-inflammatory actions independent of effects on gastric acid secretion. In esophageal cell cultures, omeprazole has been reported to inhibit Th2 cytokine-stimulated expression of eotaxin-3, an eosinophil chemoattractant contributing to esophageal eosinophilia in eosinophilic esophagitis (EoE). The objective of this study was to elucidate molecular mechanisms underlying PPI inhibition of IL-4-stimulated eotaxin-3 production by esophageal cells.

**Methods/Findings:**

Telomerase-immortalized and primary cultures of esophageal squamous cells from EoE patients were treated with IL-4 in the presence or absence of acid-activated omeprazole or lansoprazole. We measured eotaxin-3 protein secretion by ELISA, mRNA expression by PCR, STAT6 phosphorylation and nuclear translocation by Western blotting, eotaxin-3 promoter activation by an exogenous reporter construct, and STAT6, RNA polymerase II, and trimethylated H3K4 binding to the endogenous eotaxin-3 promoter by ChIP assay. Omeprazole in concentrations ≥5 µM significantly decreased IL-4-stimulated eotaxin-3 protein secretion and mRNA expression. Lansoprazole also blocked eotaxin-3 protein secretion. Omeprazole had no effect on eotaxin-3 mRNA stability or on STAT6 phosphorylation and STAT6 nuclear translocation. Rather, omeprazole blocked binding of IL-4-stimulated STAT6, RNA polymerase II, and trimethylated H3K4 to the eotaxin-3 promoter.

**Conclusions/Significance:**

PPIs, in concentrations achieved in blood with conventional dosing, significantly inhibit IL-4-stimulated eotaxin-3 expression in EoE esophageal cells and block STAT6 binding to the promoter. These findings elucidate molecular mechanisms whereby patients with Th2 cytokine-driven esophageal eosinophilia can respond to PPIs, independent of effects on gastric acid secretion.

## Introduction

For more than two decades now, proton pump inhibitors (PPIs) have been the mainstay of therapy for severe gastroesophageal reflux disease (GERD) [1]. The PPIs are potent inhibitors of H^+^,K^+^ATPase, the proton pump of the gastric parietal cell [2], and it has been assumed widely that gastric acid inhibition is the sole mechanism underlying the beneficial effects of PPIs in GERD and other acid-peptic disorders. For patients with upper gastrointestinal symptoms of uncertain etiology, such as non-ulcer dyspepsia and non-cardiac chest pain, a salutary response to PPI therapy is regarded as evidence of an underlying acid-peptic disease [3], [4]. However, PPIs have been found to have a number of anti-inflammatory actions that are independent of their effects on gastric acid secretion [5]. Conceivably, those anti-inflammatory effects of PPIs that are independent of their anti-secretory effects might contribute to the therapeutic actions of PPIs on inflammatory diseases of the gastrointestinal tract. If so, then the assumption that only acid-peptic disorders can respond to PPIs might be incorrect.

Eosinophilic esophagitis (EoE) is a chronic, immune/antigen-mediated esophageal disease characterized clinically by symptoms related to esophageal dysfunction and histologically by eosinophil-predominant inflammation [6]. Dysphagia, food impaction, and chest pain are the typical symptoms of EoE, in which esophageal biopsy specimens usually demonstrate ≥15 eosinophils per high power field, basal zone hyperplasia, and dilated intercellular spaces. These same symptoms and histological abnormalities can be found in patients with GERD, however, and occasionally it can be difficult to distinguish the two disorders [7]. In equivocal cases, an empiric trial of PPI therapy is used with the assumption that, if the symptoms and esophageal eosinophilia improve, then the patient has GERD, not EoE [8], [9]. Recently, this assumption has been called into question by the recognition of a group of patients whose esophageal symptoms and eosinophilia respond to PPIs even though they have no evidence of GERD by endoscopy or 24-hour esophageal pH monitoring [6], [10]. It is possible that patients with this “PPI-responsive esophageal eosinophilia” respond to the anti-inflammatory effects, not the anti-secretory effects, of PPIs.

The chemokine eotaxin-3 is a potent eosinophil chemoattractant that appears to play a key role in drawing eosinophils to the esophagus in EoE. In esophageal squamous cells from EoE and GERD patients, the expression of eotaxin-3 is stimulated by T helper (Th)2 cytokines such as IL-4 and IL-13, whose effects are mediated by the signal transducer and activator of transcription (STAT)6 signaling pathway [11]–[14]. Recently, we have reported that the PPI omeprazole, in a concentration of 50 µM, inhibits Th2 cytokine-induced expression of eotaxin-3 mRNA and protein by esophageal squamous cells *in vitro* [14]. Clearly, this inhibitory effect of omeprazole on esophageal squamous cells in culture cannot be due to any PPI effects on gastric acid secretion. These studies suggest that PPIs might have a role in the treatment of EoE, even if there is no associated GERD. Moreover, inhibition of cytokine-stimulated eotaxin-3 expression in the esophageal squamous epithelium is a potential explanation for the phenomenon of PPI-responsive esophageal eosinophilia. Little is known about the molecular mechanisms underlying this recently recognized, suppressive effect of PPIs on Th2 cytokine-stimulation of eotaxin-3 in the esophagus. The aim of this study was to elucidate those mechanisms in esophageal squamous epithelial cells from patients with EoE.

## Materials and Methods

### Ethics Statement

Experimental methods using human subjects were approved by the institutional review board on human studies at the Dallas VA Medical Center. Written informed consent was obtained from each subject.

### Culture of Esophageal Squamous Cells

We used two non-neoplastic, telomerase-immortalized, esophageal squamous cell lines (EoE1-T and EoE2-T) and two primary cultures of esophageal squamous cells (EoE1 and EoE2) that were created from esophageal mucosal biopsy specimens from patients who had EoE by our laboratory as previously described [14]. Briefly, the patients fulfilled the criteria for EoE suggested in the 2007 consensus recommendations [8]. They had a history of dysphagia and heartburn that had responded only partially or not at all to PPIs, and esophageal biopsy specimens showing >15 eosinophils per high power field; symptoms subsequently improved dramatically with fluticasone treatment.

Cells were maintained in monolayer culture at 37°C in humidified air with 5% CO_2_ in growth medium co-cultured with a fibroblast feeder layer as previously described [15]. For individual experiments, cells were equally seeded into collagen IV-coated wells (BD Biosciences, San Jose, CA) and maintained in growth medium.

### Cytokine Stimulation of Esophageal Squamous Cells and Omeprazole Treatments

Cells were stimulated with 10 ng/ml of IL-4 (R&D Systems, Minneapolis, MN). In earlier studies, we found that IL-4 stimulated more robust expression of exotaxin-3 than IL-13, so we selected to use IL-4 exclusively for the present study [14]. For PPI studies, omeprazole (Sigma) in concentrations of 1 to 50 µM or lansoprazole (Sigma) in concentrations of 10 and 50 µM was acid-activated in medium with pH 5.5 for 30 minutes [16]. Cells were then pre-treated for either 2 or 24 hours with PPI in medium with pH 7.4 prior to the addition of IL-4. The PPI remained in the media throughout the period of cytokine stimulation.

### Enzyme-Linked Immunosorbent Assays (ELISA) for Eotaxin-3

We performed ELISA on conditioned media after 48 hours, using commercially available ELISA kits (R&D Systems) to assess the production of eotaxin-3 by esophageal cells. Cells were pre-treated for 2 hours with acid-activated omeprazole (1–50 µM) or lansoprazole (10 µM or 50 µM) in medium with pH 7.4 prior to the addition of IL-4. Cells were stimulated with IL-4, in the presence or absence of PPI, or control medium for 48 hours. Conditioned media from esophageal cells were collected and centrifuged to remove cellular debris. Eotaxin-3 concentrations were determined using commercially available ELISA kits (R&D Systems, Minneapolis, MN) per manufacturer’s instructions. The absorbance of each well was read at 450 nm and 540 nm using a DTX 880 Multimode plate reader (Beckman Coulter). Cell count was determined, and results were expressed as pg/ml of eotaxin-3 (normalized to cell number). All assays were performed in duplicate.

### Semiquantitative and Quantitative Real-Time Polymerase Chain Reaction (PCR)

Semiquantitative and real-time PCR were performed for eotaxin-3 mRNAs in EoE cells. Cells were pre-treated for 24 hours with acid-activated omeprazole (1–50 µM) in medium with pH 7.4 prior to the addition of IL-4. Cells were stimulated with IL-4, in the presence or absence of omeprazole, or control medium for 3 hours. Total RNAs were isolated from cell lines using RNeasy Mini kit (Qiagen, Valencia, CA) per manufacturer’s instructions and quantitated by Nanophotometer (IMPLEN, Westlake Village, CA). Reverse transcription was performed using QuantiTect Reverse Transcription kit (Qiagen, Valencia, CA) per manufacturer’s instructions. The primer sequences and PCR products sizes for semiquantitative analyses were as follows: (a) Eotaxin-3 forward 5′- GGAACTGCCACACGTGGGAGTGAC-3′ and Eotaxin-3 reverse 5′-CTCTGGGAGGAAACACCCTCTCC-3′, (354 bp) and (b) GAPDH forward 5′-TCCCACCTTTCTCATCCAAG-3′ and GAPDH reverse 5′-GTCTGCAAAAGGAGTGAGGC-3, (194 bp). PCR conditions consisted of 94°C for 5 min followed by 30 cycles at 94°C for 30 s, 55°C for 30 s, and 72°C for 30 s. After amplification, PCR products were electrophoresed on 2% agarose gels and stained with ethidium bromide. GAPDH transcripts served as internal controls. In addition to conventional PCR, real-time PCR for eotaxin-3 mRNA was carried out with the StepOnePlus Real-Time PCR System and SYBR Green mix (Applied Biosystems, Foster city, CA). The primer sequences for real-time PCR were as follows: (a) Eotaxin-3 forward 5′-AACTCCGAAACAATTGTACTCAGCTG-3′ and Eotaxin-3 reverse 5′-GTAACTCTGGGAGGAAACACCCTCTCC-3′, (b) β-actin forward 5′-CATCCACGAAACTACCTTCAACTCC-3′ and β-actin reverse 5′-GAGCCGCCGAATCCACACG -3′, and (c) Cyclophilin forward 5′-CCCACCGTGTTCTTCGACAT-3′ and Cyclophilin reverse 5′-CCAGTGCTCAGAGCACGAAA-3′. β-actin and cyclophilin transcripts served as internal controls. All PCR assays were performed in at least 2 separate experiments.

### Eotaxin-3 mRNA Stability

For mRNA stability experiments, cells were pre-treated for 2 hours with omeprazole 50 µM in medium with pH 7.4 prior to the addition of IL-4. Omeprazole remained in the medium while cells were stimulated with IL-4 for 18 hours, then treated with 5, 6-dichlorobenzimidazole riboside (DRB) 50 µmol/L (Sigma) for 6, 12, 24, and 36 hours. Real-time PCR was carried out as described above.

### Nuclear/Cytoplasmic Fractionation and Western Blotting

Cells were pre-treated for 24 hours with acid-activated omeprazole (50 µM) in medium with pH 7.4 prior to the addition of IL-4. Whole cells were lysed in 1× cell lysis buffer (Cell Signaling Technology, Danvers, MA) after 20 minutes of IL-4 stimulation. Nuclear extracts were isolated after 30 minutes of IL-4 stimulation using the NE-PER Nuclear and Cytoplasmic Extraction kit (Thermo Fisher Scientific, Rockford, IL) per manufacturer’s instructions. Protein concentrations were determined using the BCA-200 Protein Assay kit (Pierce, Rockford, IL). After separation and transfer to nitrocellulose membranes, the membranes were incubated with 1∶1000 dilutions of phospho-STAT6 (Tyr641) or total STAT6 (Cell Signaling), or 1∶2000 dilutions of β-tubulin (Sigma, St. Louis, MO) or 1∶1000 dilutions of lamin A/C (Cell Signaling). Horseradish peroxidase secondary antibodies were used, and chemiluminescence was determined using the ECL Western blotting Substrate or the Super Signal West Dura detection system (Pierce). All Western blots were performed in duplicate.

### Chromatin Immune-Precipitation (ChIP) Assay

ChIP assay was performed according to the protocol published by Nelson et al. with minor modifications [17]. Cells were pre-treated for 24 hours with acid-activated omeprazole (50 µM) in medium with pH 7.4 prior to the addition of IL-4. Cells were stimulated with IL-4 in the presence or absence of omeprazole, or control medium for 1 hour. In brief, EoE cells were cross-linked with 1.4% formaldehyde for 15 min at room temperature. The reaction was stopped by treatment with 125 mM glycine for 5 min at room temperature and the cells were scraped and centrifuged to pellet. The cells were lysed in IP buffer containing 150 mM NaCl, 50 mM Tric-Hcl (pH 7.5), 5 mM EDTA, NP-40 (0.5% vol/vol), Triton X-100 (1.0% vol/vol), 0.1 M PMSF, and one Protease Inhibitor Cocktail Tablet per 50 ml of lysis buffer (Roche Applied Science, Indianapolis, IN). Following lysis, cells were centrifuged, washed, and resuspended in the IP buffer. To shear the chromatin, the resuspended pellet was sonicated to generate DNA fragments of 0.5–1 kilobase. The sheared chromatin was cleared by centrifugation and the supernatant (500 µg) was immunoprecipitated overnight at 4°C with 1.6 µg of polyclonal rabbit anti-human STAT6 (Santa Cruz) or anti-human Histone H3 trimethyl K4 (H3K4me3, Abcam) or monoclonal mouse anti-human RNA Polymerase II (Millipore); rabbit IgG or mouse IgG were used as isotype controls. Following immunoprecipitaton, the chromatin was again cleared by centrifugation and 90% of the supernatant was transferred to prewashed Protein A-agarose beads (Upstate Biotechnologies, Billerica, MA) diluted 1∶1 with IP buffer (20 µl beads:20 µl IP buffer) and rotated at 4°C for 45 min and again centrifuged to pellet. The beads were then washed 5–6 times with IP buffer without the protease inhibitors and 100 µl of 10% Chelex 100 were added followed by boiling for 10 min. The DNA was then precipitated by centrifuging the sample at 12,000 g at 4°C for 1 min, collecting the supernatant, washing the beads with 120 µl of H_2_O, followed by another centrifugation. The pooled supernatants served as the template for the PCR. For real-time and semiquantitative PCR of the pooled supernatants, 1 µl of purified DNA template was used in a 25 µl reaction with the eotaxin-3 forward primer 5′-GTGCTGCTTCTGTTCCCAACCACA-3′ and the eotaxin-3 reverse primer 5′-ACTCCTGCCTGATCCCCTT-3′ spanning nucleotides −98 to −11 within the eotaxin-3 promoter to assess for STAT6 and RNA Pol II binding and the eotaxin-3 forward primer 5′-GTTGGGTCAAAA GTGCTGCTTCTG-3′ and the eotaxin-3 reverse primer 5′-GGTGGAGACTCAGGAGGGAGGC-3′ spanning nucleotides −110 to +85 within the eotaxin-3 promoter to assess for H3K4me3 binding. Both primer sets included the proximal STAT6 binding site and the TATA box; 1% of reaction-sheared chromatin that did not undergo immunoprecipitation was used as an input control. Real-time PCR was carried out as detailed above. PCR conditions for semiquantitative analyses of H3K4me3 binding consisted of 94°C for 5 min followed by 40 cycles at 94°C for 30 s, 60°C for 30 s, and 72°C for 30 s. After amplification, PCR products were electrophoresed on 2% agarose gels and stained with ethidium bromide. All ChIP assays were performed in triplicate.

#### Eotaxin-3 promoter activity

Plasmid constructs containing the proximal 800 bp of the eotaxin-3 promoter cloned into pGL3 upstream of a luciferase reporter (EO 1) were used for the transfection studies; the renilla reporter pHRL-TK was used to equalize for transfection efficiency (EO 1 plasmid was the generous gift of Dr. Marc Rothenberg, Cincinnati Children’s Hospital, Cincinnati, OH). EO 1 contains both the distal (−693) and proximal (−89) STAT6 binding sites [11]. Cells were grow in 24-well plates and co-transfected with 500 ng of EO 1 and 25 ng of pHRL-TK using Lipofectamine LTX with Plus Reagent (Invitrogen, Carlsbad, CA) per manufacturer’s instructions. After 24 hours, cells were pre-treated for 2 hours with acid-activated omeprazole (50 µM) in medium with pH 7.4 prior to the addition of IL-4. Cells were stimulated with IL-4 in the presence or absence of omeprazole, or control medium for 24 hours. Cell extracts were then assayed for luciferase activities using the Dual-Luciferase Reporter Assay system (Promega, Madison, WI) per manufacturer’s instructions. Data were expressed as relative light units for firefly luciferase normalized to renilla luciferase. All assays were performed at least in duplicate.

### Statistical Analyses

Quantitative data are expressed as mean ± standard error of the mean (SEM). Statistical analyses were performed using an unpaired Student’s t-test with the Instat for Windows statistical software package (GraphPad Software, San Diego, CA). For multiple comparisons, ANOVA and Student-Newman-Keuls multiple-comparisons test were performed. *P* values ≤0.05 were considered significant for all analyses.

## Results

### Omeprazole Suppresses IL-4-Stimulated Eotaxin-3 Protein Secretion in Primary Esophageal Squamous Cells from EoE Patients

**Figure 1:**
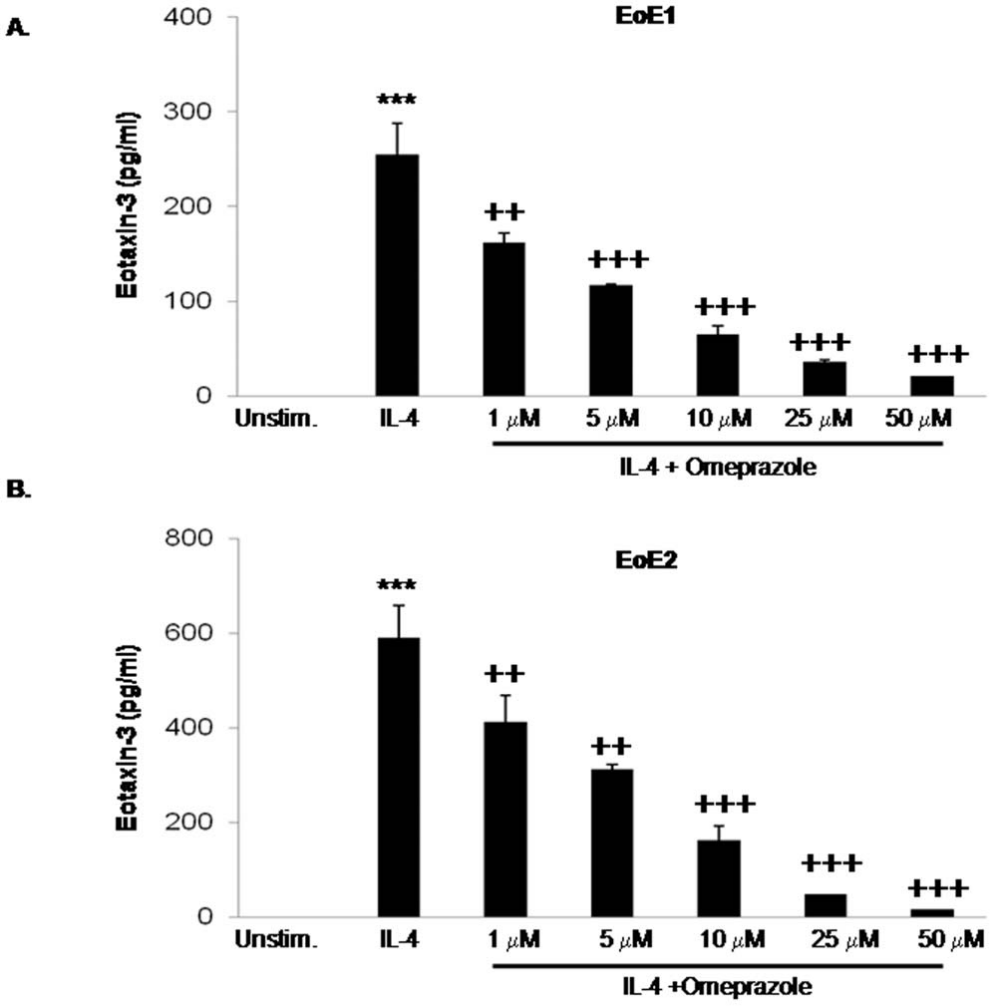
Omeprazole decreases IL-4-stimulated eotaxin-3 protein secretion in primary esophageal squamous cells from two patients with EoE, (A) EoE1 and (B) EoE2. Data are the mean ± SEM of 2 separate experiments. ***, p≤0.001 compared to unstimulated (baseline) control, ^++^, p≤0.01 compared to IL-4 stimulation alone; ^+++^, p≤0.001 compared to IL-4 stimulation alone. Unstim.; unstimulated cells.

To isolate the effect of omeprazole on esophageal epithelial cells, we studied IL-4-stimulated eotaxin-3 secretion in primary esophageal squamous cell cultures from 2 patients with EoE. Stimulation with IL-4 for 48 hours caused a significant increase in eotaxin-3 protein secretion in both EoE primary cell cultures (Figure 1). Treatment with 1, 5, 10, 25 and 50 µM doses of omeprazole significantly decreased the stimulated eotaxin-3 protein secretion in both EoE primary cell cultures (Figure 1).

### Both Omeprazole and Lansoprazole Suppress IL-4 Stimulated Eotaxin-3 Protein Secretion in Esophageal Squamous Cell Lines from EoE Patients

**Figure 2:**
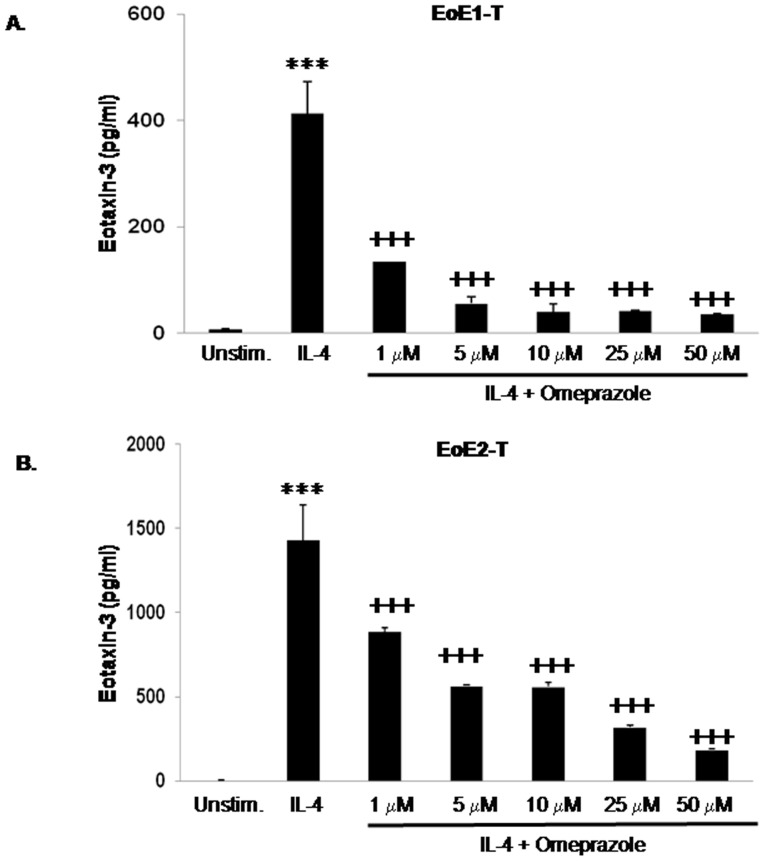
Omeprazole decreases IL-4-stimulated eotaxin-3 protein secretion intelomerase-immortalized esophageal squamous cell lines from two patients with EoE, (A) EoE1-T and (2) EoE2-T. Data are the mean ± SEM of 2 separate experiments. ***, p≤0.001 compared to unstimulated (baseline) control, ^+++^, p≤0.001 compared to IL-4 stimulation alone. Unstim.; unstimulated cells.

Using our telomerase-immortalized cell lines established from EoE1 and EoE2 primary cell cultures, we explored the mechanisms underlying the inhibitory effect of PPIs on IL-4-stimulated eotaxin-3 production. There were no significant differences between untreated control cells and cells treated with 50 µM omeprazole alone in their baseline levels of eotaxin-3 protein section (p>0.05 in both EoE1-T and EoE2-T cells). As in our primary cell cultures, stimulation with IL-4 for 48 hours caused a significant increase in eotaxin-3 secretion (Figure 2). Treatment with 1, 5, 10, 25 and 50 µM doses of omeprazole significantly decreased eotaxin-3 protein secretion in both EoE cell lines (Figure 2).

**Figure 3:**
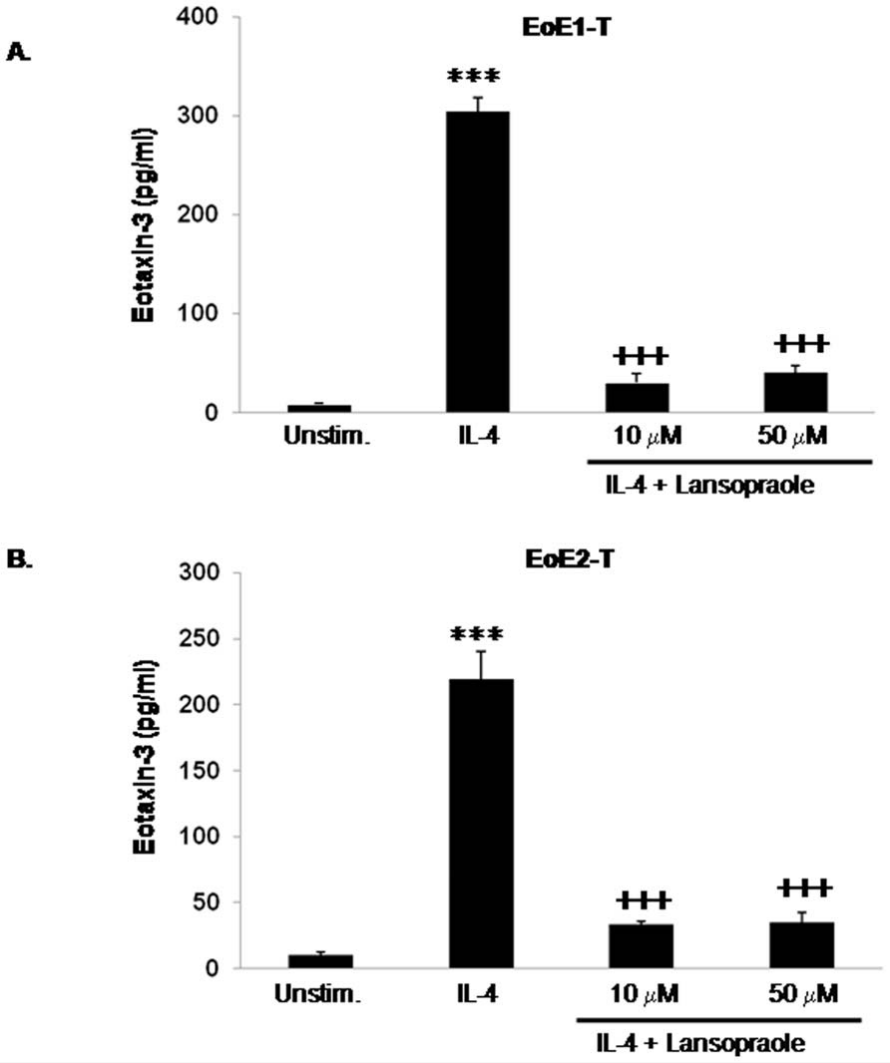
Lansoprazole decreases IL-4-stimulated eotaxin-3 protein secretion in (A) EoE1-T and (2) EoE2-T cells. Data are the mean ± SEM of at least 2 separate experiments. ***, p≤0.001 compared to unstimulated (baseline) control, ^+++^, p≤0.001 compared to IL-4 stimulation alone. Unstim., unstimulated cells.

We next studied effects of lansoprazole on IL-4 stimulated eotaxin-3 protein secretion to determine if the inhibitory effect we had observed for omeprazole was unique to that drug among the PPIs. We found no significant differences between untreated control cells and cells treated with 50 µM lansoprazole alone in their baseline levels of eotaxin-3 protein section (p>0.05 in both EoE1-T and EoE2-T cells). Like omeprazole, lansoprazole in 10 µM and 50 µM concentrations significantly decreased IL-4-stimulated eotaxin-3 protein secretion in both EoE cell lines (Figure 3). These findings suggest that inhibition of IL-4-stimulated eotaxin-3 protein secretion by esophageal squamous cells is not unique to omeprazole, and might be a class effect of PPIs.

### Omeprazole Suppresses IL-4-Stimulated Eotaxin-3 mRNA Expression in EoE Cell Lines

**Figure 4:**
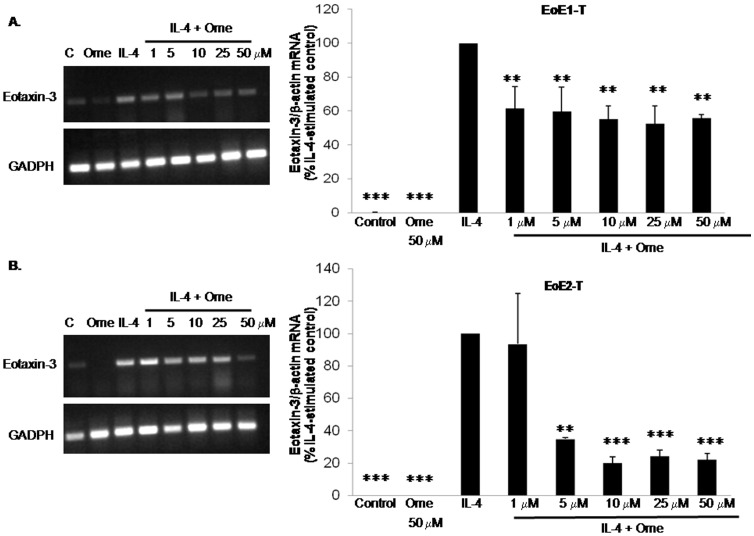
Omeprazole (Ome) decreases IL-4-stimulated eotaxin-3 mRNA expression levels in (A) EoE1-T and (B) EoE2-T cell lines as determined by conventional PCR and quantitative real-time PCR. Depicted is of one of at least 2 separate experiments. **, p≤0.01 compared to IL-4 stimulation alone; ***, p≤0.001 compared to IL-4 stimulation alone. C, unstimulated control cells.

To determine whether omeprazole affected IL-4-induced eotaxin-3 transcriptional regulation, RT-PCR and real-time PCR were performed to evaluate eotaxin-3 mRNA expression. We treated the cells with IL-4 in the presence of 1–50 µM concentrations of omeprazole. Compared to cells stimulated with IL-4 alone, we found a significant decrease in eotaxin-3 mRNA levels for all concentrations of omeprazole in EoE1-T (Figure 4A), and for omeprazole in concentrations ≥5 µM in EoE2-T cells (Figure 4B).

### Omeprazole Does Not Enhance Eotaxin-3 mRNA Degradation in EoE Cells

**Figure 5:**
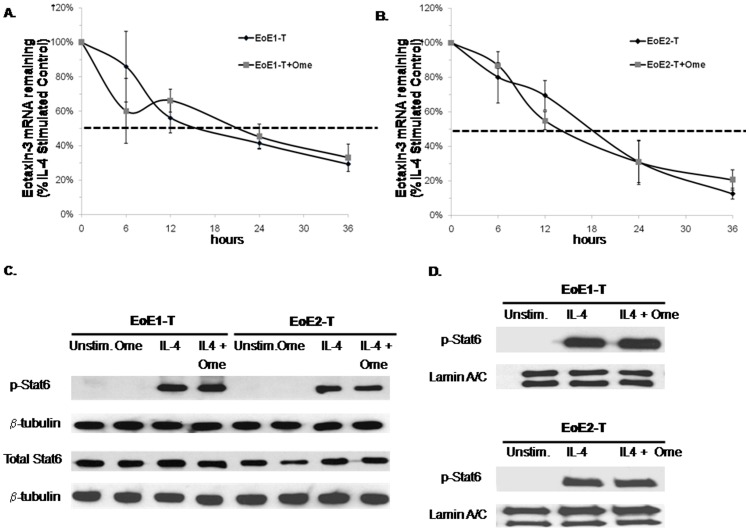
Omeprazole (Ome) does not increase IL-4-stimulated eotaxin-3 mRNA degradation, and does not decrease STAT6 phosphorylation or nulcear translocation in EoE1-T and EoE2-T cell lines. mRNA expression levels were determined by quantitative real-time PCR in (A) EoE1-T and (B) EoE2-T cells. Data are the means ± SEM of 3 separate experiments assayed in triplicate. Representative experiments of Western blotting for (C) phospho- and total STAT6 in whole cell lysates and (D) phospho-STAT6 in nuclear lysates in EoE1-T and EoE2-T cells. Tubulin and lamin A/C served as controls for the whole cell and nuclear lysates, respectively. Unstim., unstimulated cells.

Possible explanations for the marked decrease in IL-4-stimulated eotaxin-3 mRNA levels that we observed with omeprazole treatment of our EoE cells included an omeprazole-induced decrease in mRNA transcription, an omeprazole-induced increase in mRNA degradation, or both. To explore effects of omeprazole on mRNA degradation, EoE cells were treated with both omeprazole and DRB, an inhibitor of mRNA synthesis. For both EoE1-T and EoE2-T, there were no significant differences between omeprazole-treated and untreated cells in the percentage of decline in IL-4 stimulated eotaxin-3 mRNA levels at any time point (p>0.05) (Figure 5A&B). These findings suggest that omeprazole does not cause an increase in degradation of eotaxin-3 mRNA in EoE cells.

### Omeprazole Does Not Inhibit STAT6 Phosphorylation or Nuclear Translocation in EoE Cells

In earlier studies, we showed that IL-4 induced transcriptional regulation of eotaxin-3 mRNA expression is mediated by STAT6 signaling in esophageal squamous cells from EoE patients and, in a number of other epithelial cell types, PPIs have been found to inhibit STAT6 phosphorylation [14], [16]. Therefore, we performed Western blots for STAT6 phosphorylation in the presence and absence of 50 µM of omeprazole. There were no apparent differences in IL-4 stimulated phosphorylation levels for STAT6 between omeprazole treated and untreated EoE cells (Figure 5C). Upon phosphorylation and activation, STAT6 dimerizes and translocates to the nucleus where it can bind DNA and activate transcription of target genes [18]. Therefore, we explored whether omeprazole interfered with the ability of phospho-STAT6 to undergo nuclear translocation. Western blot revealed no apparent differences in IL-4-stimulated nuclear expression levels of phospho-STAT6 between omeprazole treated and untreated EoE cells (Figure 5D). These findings suggest that omprazole does not inhibit STAT6 phosphorylation or nuclear translocation in EoE cells.

### Omeprazole Reduces IL-4 Stimulated Binding of STAT6 to the Endogenous Eotaxin-3 Promoter in EoE Cells

**Figure 6:**
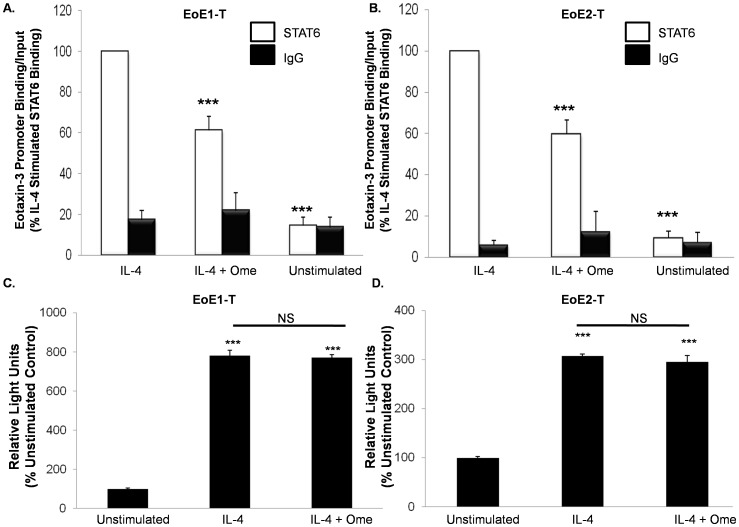
Omeprazole (Ome) decreases IL-4-stimulated STAT6 binding to the endogenous eotaxin-3 promoter in (A) EoE1-T and (B) EoE2-T cells. Data are the means ± SEM of 3 separate experiments. ***, p≤0.001 compared to IL-4 stimulation. Isotype matched IgG served as a control. Omeprazole does not inhibit activation of the transiently transfected, exogenous, eotaxin-3 EO1 promoter (−800 bp) construct in (C) EoE1-T and (D) EoE2-T cells. Data are the mean ± SEM of 2 separate experiments. ***, *p*<0.001 compared to unstimulated control cells.

We used a ChIP assay to determine whether omeprazole interfered with STAT6 binding to the eotaxin-3 promoter; isotype matched IgG served as a negative control. Omeprazole significantly decreased binding of IL-4-stimulated STAT6 to the eotaxin-3 promoter in EoE1-T and EoE2-T cells (Figure 6A&B). Two possible explanations for this finding are: 1) omeprazole induces modifications to the eotaxin-3 chromatin structure or 2) omeprazole induces modifications of the STAT6 protein that block its functional activities (i.e. DNA binding and/or activation of its transcriptional complex). To distinguish between these possibilities, we performed transient transfections with the full length EO1 (−800 bp) promoter construct. In earlier studies using our EoE cell lines, we showed that IL-4-stimulated activation of this exogenous promoter construct requires STAT6 binding [11], [14]. Unlike endogenous promoters, transiently transfected promoters have an open and accessible chromatin structure, and they are not subject to complex chromatin regulatory mechanisms [19]. Also, transcriptional activation of the promoter usually requires binding of a set of transcriptional co-regulatory proteins to the TAD domain of STAT6 [18], [20]. For both EoE1-T and EoE2-T, we found no significant differences between omeprazole-treated and untreated cells in the degree of activation of the exogenous eotaxin-3 promoter (Figure 6C&D). These findings show that omeprazole does not induce modifications to the STAT6 protein that interfere with its function and, by inference, suggest that omeprazole might alter eotaxin-3 chromatin.

### Omeprazole Reduces IL-4-Stimulated Binding of RNA Pol II to the Endogenous Eotaxin-3 Promoter in EoE Cells

**Figure 7:**
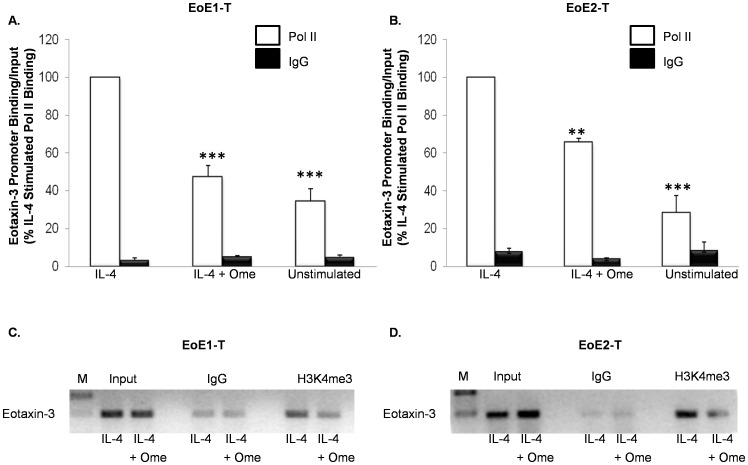
Omeprazole (Ome) decreases IL-4-stimulated RNA Pol II binding to the endogenous eotaxin-3 promoter in (A) EoE1-T and (B) EoE2-T cells. Data are the means ± SEM of 3 separate experiments. **, p≤0.01 compared to IL-4 stimulation; ***, p≤0.001 compared to IL-4 stimulation Isotype matched IgG served as a control. Representative experiment demonstrating that omeprazole reduces the levels of IL-4 stimulated H3K4me3 bound to the endogenous eotaxin-3 promoter in (C) EoE1-T and (D) EoE2-T cells. Isotype matched IgG served as a control. Depicted is one of 3 separate experiments. M, marker.

To address whether the omeprazole-induced decrease in binding of STAT6 was accompanied by decreased transcriptional activity, RNA polymerase II (Pol II) binding to the eotaxin-3 promoter was assessed by ChIP assay; isotype matched IgG served as a negative control. Omeprazole significantly decreased binding of IL-4-stimulated Pol II to the endogenous eotaxin-3 promoter in EoE1-T and EoE2-T cells, a finding consistent with a reduction in eotaxin-3 transcriptional activity (Figure 7A&B). Trimethylation of the lysine 4 in histone H3 (H3K4me3) is thought to be a post-translational modification of histone H3 that occurs as genes are induced, and approximately 91% of all RNA Pol II binding sites correlate with H3K4me3 binding sites [21]–[23]. Using ChIP assay, we found that omeprazole reduced the levels of IL-4-stimulated H3K4me3 bound to the endogenous eotaxin-3 promoter in EoE1-T and EoE2T cells (Figure 7C&D). These data suggest that omeprazole causes chromatin remodeling in the eotaxin-3 promoter, resulting in decreased RNA Pol II recruitment and reduced eotaxin-3 transcriptional activity in EoE cells.

## Discussion

In primary and telomerase-immortalized esophageal squamous cells from patients with EoE, we have shown that omeprazole, in concentrations as low as 1 µM, significantly inhibits IL-4-stimulated eotaxin-3 protein expression. We also have demonstrated that this inhibition appears to be a class effect of PPIs, because lansoprazole in low concentration (10 µM) also decreases cytokine-stimulated eotaxin-3 protein expression in EoE squamous cell lines. We have shown that omeprazole reduces IL-4-stimulated eotaxin-3 mRNA expression, and that this effect is not due to enhanced mRNA degradation, reduced STAT6 phosphorylation, or decreased phospho-STAT6 nuclear translocation. Rather, we have found that omeprazole blocks IL-4-stimulated mRNA transcription by reducing the binding of STAT6 and RNA polymerase II to the eotaxin-3 promoter, effects which are not due to alterations in the functional activity of STAT6 protein. We also have found that omeprazole reduces the levels of IL-4-stimulated H3K4me3 bound to the eotaxin-3 promoter. These findings suggest that, in EoE cells, omeprazole causes chromatin remodeling in the eotaxin-3 promoter, resulting in decreased RNA Pol II recruitment and reduced eotaxin-3 transcriptional activity. Thus, our findings elucidate molecular mechanisms whereby patients with Th2 cytokine-induced esophageal eosinophilia can respond to PPIs.

Although PPIs are prescribed primarily with the intent of controlling gastric acid secretion, these agents have been found to have a number of potentially beneficial biological actions that are independent of their antisecretory effects. For example, PPIs have demonstrable anti-oxidant properties, and have been found to inhibit certain neutrophil functions, to decrease adhesion molecule production and to block the production of the pro-inflammatory cytokine IL-8 by endothelial and epithelial cells [reviewed in [5]]. In xenograft models, furthermore, PPIs decrease the growth of human tumors, presumably as a result of inhibiting the tumor cells’ vacuolar-ATPase (V-ATPase), a proton pump that regulates intra- and extracellular pH [24]. In the present study, we have elucidated the molecular mechanisms underlying another gastric acid-independent, potentially beneficial PPI effect, namely the inhibition of Th2 cytokine-stimulated eotaxin-3 production by esophageal squamous cells.

In an earlier, “proof of principle” study, we found that omeprazole in high concentration (50 µM) decreased Th2 cytokine-stimulated eotaxin-3 expression by esophageal squamous cells [14]. With conventional oral dosing of omeprazole, peak mean plasma concentrations of 3.2 µM have been documented, whereas levels as high as 10 µM have been reported with intravenous administration [25], [26]. In the present study, we found significant inhibition of IL-4-stimulated eotaxin-3 protein expression in esophageal squamous cells treated with omeprazole in concentrations as low as 1 µM. Thus, we have documented that the inhibitory effect of omeprazole on Th2 cytokine-stimulated eotaxin-3 expression occurs *in vitro* using concentrations of the drug that are readily achieved in blood with conventional oral dosing.

For our experiments, we used acid-activated omeprazole and lansoprazole, and it is not clear whether acid activation of PPIs can occur in the esophagus. PPIs are known to accumulate and become activated in an acidic tissue microenvironment, which is found frequently around gastric parietal cells. Immune cells, including eosinophils and neutrophils, also can release protons from their exocytic granules and lysosomes into the microenvironment [27]–[29] and, in the setting of inflammation associated with infection, asthma, and rheumatoid arthritis, microenvironmental acidification has been documented [30]–[32]. Gastresophageal acid reflux also might acidify the esophageal microenvironment, and the Na^+^/H^+^ exchanger on esophageal epithelial cell membranes is known to extrude intracellular protons that accumulate in the setting of injury in order to maintain intracellular pH [33]. Thus, there are a number of plausible mechanisms whereby PPIs might be activated in an acidic microenvironment of the esophagus that is inflamed by EoE or GERD.

In an earlier study, we showed that IL-4-stimulated eotaxin-3 expression in esophageal squamous cells is mediated by STAT6 signaling [14], and there are a number of points in that signaling pathway where omeprazole might exert its inhibitory effects. In the stomach, omeprazole is known to alkylate cysteine residues in gastric H^+^,K^+^ATPase. In epithelial and non-epithelial cells, Cortes *et al.* found that omeprazole decreased Th2 cytokine-induced STAT6 phosphorylation, an effect that they attributed to the PPI’s alkylating ability [16]. Omeprazole did not affect total STAT6 protein levels in that study, and the authors suggested that the decreased STAT6 phosphorylation might be due to omeprazole-induced modifications either of the STAT6 protein itself or of the ability of upstream kinases to phosphorylate the protein [16]. In murine myeloid cells, Perez *et al.* described a different mechanism by which the drug n-alpha-tosyl-L-phenylalanine-chloromethyl ketone (TPCK) interfered with IL-4-induced STAT6 activation [34]. By virtue of its alkylating properties, TPCK induced modifications of the STAT6 protein’s cysteine residues, which facilitated the degradation of total STAT6, thus resulting in decreased STAT6 phosphorylation levels [34]. In contrast to these studies, we found no apparent effect of omeprazole on the levels of IL-4-induced STAT6 phosphorylation or total STAT6 protein in our EoE cells. Moreover, we found no apparent effect of omeprazole on the nuclear translocation of phospho-STAT6.

Our observation that omeprazole significantly decreases the binding of STAT6 to the eotaxin-3 promoter suggests either that omeprazole induces modifications to the eotaxin-3 chromatin structure, or that omeprazole induces modifications of the STAT6 protein that affect its function. To distinguish between these possibilities, we transiently transfected with an exogenous eotaxin-3 promoter construct that is activated by STAT6 binding [11], [14]. Unlike the endogenous promoter. this exogenous eotaxin-3 promoter has an open and accessible chromatin structure, and is not subject to complex chromatin regulatory mechanisms [19]. We found no significant differences between omeprazole-treated and untreated EoE cells in the degree of activation of the exogenous eotaxin-3 promoter, showing that omeprazole does not induce modifications to the STAT6 protein that interfere with its function.

To address whether the omeprazole-induced decrease in binding of STAT6 was accompanied by decreased transcriptional activity, we assessed RNA polymerase II (Pol II) binding to the eotaxin-3 promoter by ChIP assay. Our observation that omeprazole significantly decreased binding of IL-4-stimulated Pol II to the endogenous eotaxin-3 promoter suggests that the PPI reduced eotaxin-3 transcriptional activity. Using ChIP assay, we also found that omeprazole reduced the levels of IL-4-stimulated H3K4me3 bound to the endogenous eotaxin-3 promoter. This trimethylation is a post-translational modification of histone H3 that occurs as genes are induced, and approximately 91% of all RNA Pol II binding sites correlate with H3K4me3 binding sites [21]–[23]. Taken together, our observations suggest that omeprazole causes chromatin remodeling in the eotaxin-3 promoter, resulting in decreased RNA Pol II recruitment and reduced eotaxin-3 transcriptional activity in EoE cells.

In conclusion, we have shown that omeprazole, in concentrations that can be achieved in plasma with conventional dosing, significantly decreases IL-4-stimulated eotaxin-3 expression in esophageal squamous cells from patients with EoE. In those cells, omeprazole does not inhibit STAT6 phosphorylation, does not reduce the level of total STAT6 protein, and does not reduce the translocation of phopho-STAT6 to the nucleus. Rather, omeprazole reduces the transcription of eotaxin-3 mRNA by reducing the binding of STAT6 and RNA polymerase II to the exotaxin-3 promoter in association with a reduction in the levels of promoter-bound H3K4me3. These findings elucidate molecular mechanisms whereby patients with Th2 cytokine-driven esophageal eosinophilia can respond to PPIs, independent of effects on gastric acid secretion. These findings might explain the phenomenon of PPI-responsive esophageal eosinophilia, and suggest that even patients who have EoE without GERD might benefit from PPI therapy. Nevertheless, further studies are needed to establish that these PPI effects observed in cells *in vitro* are applicable to patients.
